# Disproportionate Fetal Growth and the Risk for Congenital Cerebral Palsy in Singleton Births

**DOI:** 10.1371/journal.pone.0126743

**Published:** 2015-05-14

**Authors:** Elani Streja, Jessica E. Miller, Chunsen Wu, Bodil H. Bech, Lars Henning Pedersen, Diana E. Schendel, Peter Uldall, Jørn Olsen

**Affiliations:** 1 Department of Epidemiology, School of Public Health, University of California Los Angeles, Los Angeles, California, United States of America; 2 Department of Obstetrics and Gynecology, Institute of Clinical Medicine, Aarhus University Hospital, Aarhus, Denmark; 3 Section of Epidemiology, Department of Public Health, Aarhus University, Aarhus, Denmark; 4 National Centre for Register-based Research, Department of Economics and Business, Aarhus University, Aarhus, Denmark; 5 Lundbeck Foundation Initiative for Integrative Psychiatric Research, iPSYCH, Aarhus, Denmark; 6 The Danish Cerebral Palsy Registry, National Institute of Public Health, Southern University, Copenhagen, Denmark; 7 Pediatric Department, Rigshospitalet, University of Copenhagen, Copenhagen, Denmark; Hôpital Robert Debré, FRANCE

## Abstract

**Objective:**

To investigate the association between proportionality of fetal and placental growth measured at birth and the risk for congenital cerebral palsy (CP).

**Study Design:**

We identified all live-born singletons born in Denmark between 1995 and 2003 and followed them from 1 year of age until December 31^st^, 2008. Information on four indices of fetal growth: ponderal index, head circumference/ abdominal circumference ratio, cephalization index and birth weight/ placenta weight ratio was collected. Cox proportional hazards regression models were used to estimate adjusted hazard ratios (aHR) and 95% confidence intervals (CI). All measurements were evaluated as gestational age and sex specific z-scores and in z-score percentile groups, adjusted for potential confounders, and stratified on gestational age groups (<32, 32-36, 37-38, 39, 40, ≥41 weeks).

**Results:**

We identified 503,784 singleton births, of which 983 were confirmed cases of CP. Head/ abdominal circumference ratio (aHR:1.12; 95%CI:1.07-1.16) and cephalization index (aHR:1.14; 95%CI:1.11-1.16) were associated with the risk of CP irrespective of gestational age. Birth weight-placental weight ratio was also associated with CP in the entire cohort (aHR:0.90; 95%CI:0.83-0.97). Ponderal index had a u-shaped association with CP, where both children with low and high ponderal index were at higher risk of CP.

**Conclusions:**

CP is associated with disproportions between birth weight, birth length, placental weight and head circumference suggesting pre and perinatal conditions contribute to fetal growth restriction in children with CP.

## Introduction

Congenital cerebral palsy (CP) is the most common physical developmental disability in children occurring with a birth prevalence of 2 per 1000 live births[1, 2]. The pathogenesis of CP has been attributed to antenatal as well as intrapartum factors. Intrauterine growth restriction induced by antenatal conditions and resulting in low birth weight for gestational age is one of the main risk indicators[3].

Disproportionate or “asymmetric” fetal growth has been defined by abnormal ratios of anthropometric measurements at birth for a given gestational age. Cox el al[4] have defined asymmetric growth as a later onset of reduced body weight for gestational age associated with atrophic internal organs and high brain/liver ratio. Similarly, Nardozza el al[5] describe asymmetric growth as a late onset disproportion, with the fetal length and head circumference corresponding more to gestational age than to weight or abdominal circumference. The fetal weight-placental weight ratio is another clinically significant measurement that has been used to describe fetal disproportional growth [6].

Fetal growth abnormalities, which may include both symmetric or asymmetric growth, may be induced by a wide variety of antenatal factors including potential risk factors for CP such as placental abnormalities[7–9], parental age[10–17], smoking during pregnancy[18–20], socio-economic status[21–23], child sex[19, 24], first liveborn[11, 25] and calendar year of birth[1, 26] and maternal medical conditions in pregnancy such as hypertensive disorders of pregnancy[11, 27], vaginal bleeding during pregnancy[28, 29] and maternal type 1-diabetes[11],[30]. Previous studies on the association between CP and newborn anthropometric measurements have mostly been limited to analyses of birth weight for a given gestational age. Associations of traditional indexes of asymmetric growth with CP have received little attention. Although studies based on anthropometric measurements without imaging cannot differentiate between various causes of asymmetric growth and organ size, such studies may direct future studies further examining CP etiology. We have undertaken a population-based cohort study of the risk of CP associated with anthropometric measurements at birth, placenta weight, and their ratios, after considering important confounding factors.

## Methods

### Ethics Statement

This study was approved by the Danish Data Protection Agency 2010-41-4543 on December 14th, 2011, Research Ethic Committee and University of California, Los Angeles Institutional Review Board. Informed Consent was waived because information involving the subjects was recorded in such a manner that the subject could not be identified directly or through identifiers linked to the subject by the investigator.

### Study Population

In Denmark’s comprehensive registration systems, all residents in Denmark are assigned a unique personal identification number at birth, which enables linkage of individual information among all Danish national registries[31]. The Danish Medical Birth Register contains data on all live births and stillbirths in Denmark, including characteristics of mother and child related to pregnancy and delivery[32]. In 1997, additional perinatal factors were added to the registry such as placenta weight. We identified all live-born singletons born in Denmark between January 1, 1996 and December 31, 2003. Of these, 503,784 were alive and did not emigrate from Denmark before one year after birth and were used in the analysis (S1 Fig). Additional information about the registries is available at www.dst.dk.

### Congenital Cerebral Palsy

Children were identified as having *validated CP* if they were alive after the first year of life and included in the Danish National Cerebral Palsy Register. The Danish National Cerebral Palsy Register is a population-based registry that contains a record of individuals with validated CP diagnosis[33]. Data are collected from pediatric departments and other hospital records. Records from CP cases are validated by a child neurologist and an obstetrician based on review of the child’s physical findings recorded in medical records and information is then registered in a standard form[33]. In order for a child to be included in the register as a CP case, the child must have survived and lived in Denmark till age 5–6 years, so that the disease can be monitored and evaluated for progression. In addition, the condition must have a pre or perinatal aetiology (events occurring before 28 days), and also fulfill diagnostic criteria for CP including results of a cerebral computed tomographic scan or magnetic resonance image. Although CP cases are confirmed when the child undergoes a neurological exam at age 5–6 years, the registries report the date of diagnosis as when symptoms were first noted for the child and the parents were informed of their child’s CP condition. Therefore, time of onset of CP for the analysis was defined as the first recorded date of diagnosis in the Danish National Cerebral Palsy Register. If the child’s date of diagnosis was prior to age 1 (n = 485) or missing (n = 21), the child’s date of diagnosis was re-centered to the date of the child’s 1^st^ birthday.

### Perinatal and Postnatal Factors

Information on length of gestation(weeks), birth weight(g), birth length(cm) and, starting in 1997, placenta weight(g), abdominal circumference(cm), and head circumference(cm) all measured at birth was obtained from the Danish Medical Birth Register. Information on gestational age at birth registered in the Danish Medical Birth Register was estimated from last menstrual period and adjusted if needed by ultrasound measures during early pregnancy[34]. Information on child sex at birth, maternal smoking, maternal age, and paternal age were also obtained from the Danish Medical Birth Register. We obtained information on each parent’s highest level of completed education or training at year of birth of the child from the Danish Civil Registration System database[31]. We used the highest education level from either parent as a marker of socio-economic status (low, middle, high). Low is equivalent to high school education or less, middle is equivalent to college or vocational training, and high is equivalent to graduate level education.

### Hospital Reported Diseases

Data on maternal pregnancy related comorbidities as diagnosed in a hospital, either inpatient or outpatient, were obtained from the Danish National Hospital Register. The Danish National Hospital Register holds information on all discharges from Danish hospitals and outpatient treatments for the time of the study. Diagnostic information is based on the Danish version of the *International Classification of Diseases* 10^th^ Revision (ICD-10) and reported to the register after each hospital visit.

We categorized mother as having a maternal comorbidity if the mother was recorded in the Danish National Hospital Register with an ICD-10 code for that disease (S1 Table) between 294 days before and up to 10 days after date of birth of the child.

### Definition of variables studied

In addition to studying associations of anthropometric measurements measured at birth with CP: birth weight, placental weight, birth length, head circumference, and abdominal circumference, we also studied associations of ratios of these measurements with CP. These ratios include: ponderal index[35] (birth weight(g)/[birth length(cm)]^3^), head-abdominal circumference ratio[36], cephalization index (head circumference(cm)/birth weight(g)) and birth weight-placenta weight index[37].

Child sex and gestational age specific Z-scores [(actual value- population mean)/standard deviation for population mean)] based on the non-CP population were calculated for all these measurements and ratios and used in the analysis.

### Statistical Analysis

Characteristics of children in our cohort stratified by CP vs non-CP were analyzed as proportions, means ± standard deviations (SD). All children were followed from one year of age until a reported diagnosis of CP in the Danish National Cerebral Palsy Register, death, emigration, or December 31, 2008, whichever occurred first, as a function of exposure to anthropometric measurements measured at birth (birth weight, birth length, head circumference, abdominal circumference, placental weight) and indices of growth proportionality (ponderal index, cephalization index, head-abdominal circumference ratio, and birth weight-placenta ratio). Exposures were evaluated both as continuous z-scores and in groups of percentile z-score (≤10, >10–25,> 25–75 (reference), >75–90, >90). We additionally examined these associations in strata of gestational age (<32, 32–36, 37–38, 39, 40, ≥41 weeks). Hazard ratios (HR) and 95% confidence intervals (CI) were estimated by Cox proportional hazard regression models using PROC PHREG in SAS version 9.2[38]. Adjusted hazard ratios (aHR) included adjustment for maternal age (<20, 20–25, 25–30, 30+), paternal age (<25 25–30, 30–35, 35+), maternal smoking during pregnancy (yes, no), first liveborn (yes, no), parents’ education (low, middle, high), child’s year of birth, and presence of maternal disorders of pregnancy: vaginal bleeding, diabetes in pregnancy, hypertensive disorders during pregnancy and placenta disorders. Placenta disorders included placenta previa, abruption placentae and a few other placental disorders reported to be associated with CP. The ICD codes used to identify all maternal disorders are reported in the S1 Table.

Multiple imputation methods (“PROC MI and PROC MIANALYZE” in SAS 9.2[38]) were used to replace missing covariate data. The procedure generates 5 different simulated completed datasets, replacing each missing value with a set of plausible values based on the other available values for that variable and other covariate data. The multiply imputed data sets are then analyzed by using standard procedures for complete data and combining the results from these analyses. Missing values requiring imputation included: parents’ education (n = 194,925), maternal smoking during pregnancy (n = 21,982), paternal age (n = 11,412), maternal age (n = 3), and first liveborn (n = 4).

Because of the high number of missing values for education, smoking, and paternal age, and the uncertainty of the randomness of missing data, we additionally conducted sensitivity analyses using complete participant data analyses, in which only cohort members with complete data on all covariates were included. In order to check proportionality assumptions for the use of cox models, plots of log [-log (survival rate)] and Schoenfeld Residuals against log (survival time) were used (survival refers to non-CP diagnosis). All statistical analyses were conducted with SAS version 9.2[38].

## Results

We identified 503,784 singleton newborns that survived or did not emigrate prior to 1 year of age (date of birth+365). Among these, there were 983 confirmed cases of CP. The characteristics of the subjects are presented in Table 1. CP infants were more likely to be males, first births, born to mothers who smoked during their pregnancy, and to parents with a low combined parental education level. They also had lower average gestational age, birth weight, and placenta weight. Mothers of CP infants were more likely to have at least one of the selected disorders of pregnancy.

**Table 1:** Pregnancy characteristics of the cohort according to the presence of CP in the infant.

	ALL		NO CP		CP	
	N = 503,784		N = 502,801		N = 983	
Variable	N*	mean±SD	N*	mean±SD	N*	mean±SD
**Preterm (%)**	500,361	4.7	499,386	4.7	975	29.0
**Birth weight (g)**	499,518	3,539±567	498,565	3,540±566	953	2,913±998
**Placental weight (g)**	418,841	663±147	418,069	663±147	772	586±179
**Child Gender (% female)**	503,784	48.7	502,801	48.7	983	40.5
**First Liveborn (%)**	503,780	43.0	502,797	42.9	983	51.4
**Maternal Age (%)**	503,781		502,798		983	
** <20 years**		1.7		1.7		2.2
** 20 to 25 years**		13.7		13.7		14.4
** 25 to 30 years**		36.6		36.6		33.2
** 30 to 35 years**		33.7		33.7		34.4
** ≥35 years**		14.3		14.3		15.8
**Paternal Age (%)**	492,372		491,411		961	
** <25 years**		6.8		6.8		9.1
** 25 to 30 years**		27.0		27.0		23.5
** 30 to 35 years**		36.8		36.8		36.6
** 35 to 40 years**		20.2		20.2		21.9
** ≥40 years**		9.2		9.2		8.9
**Parents’ Education (%)**	308,859		308,238		621	
** Low**		20.0		20.0		22.1
** Middle**		39.9		39.9		41.1
** High**		40.1		40.1		36.8
**Smoking**	481,802		480,901		901	
** Smoking—NO (%)**		77.3		77.3		71.3
** Smoking—YES (%)**		22.7		22.7		28.7
**Maternal Pregnancy Disorders**	503,784		502,801		983	
** Vaginal Bleeding (%, [n])**		1.5 [7,730]		1.5 [7,692]		3.9 [38]
** Gestational Diabetes (%, [n])**		1.2 [6,193]		1.2 [6,166]		2.8 [27]
** Hypertensive Disorders of Pregnancy (%, [n])**		3.3 [16,645]		3.3 [16,578]		6.8 [67]
** Placental Disorders (%, [n])**		4.1 [20,787]		4.1 [20,716]		7.2 [71]

CP: congenital cerebral palsy, SD: standard deviation

Median z-scores for cephalization index and head-abdominal circumference ratio were higher in CP infants, while median z-scores were lower in CP infants vs. non-CP infants for all other measures (S2 Table). In fully adjusted models, continuous z-scores for all anthropometric measurements were significantly and negatively associated with CP with HRs varying between 0.72 and 0.85 (Table 2). Sensitivity analyses for Table 2 associations using complete participant data analysis showed similar results (S3 Table).

**Table 2:** Hazard Ratios (HR) for CP according to newborn anthropometric measures and indices (all subjects).

	N without CP	N with CP	HR	aHR*	95%CI
**Birth weight**	498,565	953	0.69	0.72	(0.68, 0.77)
**Birth length**	494,903	856	0.76	0.77	(0.73, 0.82)
**Head Circumference**	424,631	646	0.78	0.79	(0.74, 0.85)
**Abdominal Circumference**	411,266	577	0.71	0.73	(0.67, 0.79)
**Placental Weight**	418,069	772	0.84	0.85	(0.79, 0.92)
**Ponderal Index**	494,844	855	0.92	0.94	(0.87, 1.02)
**Cephalization Index**	423,501	643	1.15	1.14	(1.11, 1.17)
**Head-Abd. Circ. Ratio**	410,775	570	1.12	1.12	(1.07, 1.17)
**Birth weight/placenta ratio**	416,691	755	0.88	0.90	(0.83, 0.97)

CP: congenital cerebral palsy, HR: Hazard Ratio, aHR: adjusted hazard ratio, CI: confidence interval, Head-Abd. Circ. Ratio: Head-Abdominal Circumference Ratio

All exposures were analyzed as sex and gestational adjusted z-scores.

Models were adjusted for maternal age, paternal age, smoking, first liveborn, parents’ education, year of child’s birth,

vaginal bleeding, diabetes in pregnancy, hypertensive disorder during pregnancy and placenta disorders.

### Birth Weight, Birth Length, Abdominal Circumference, and Placenta Weight

**Fig 1:**
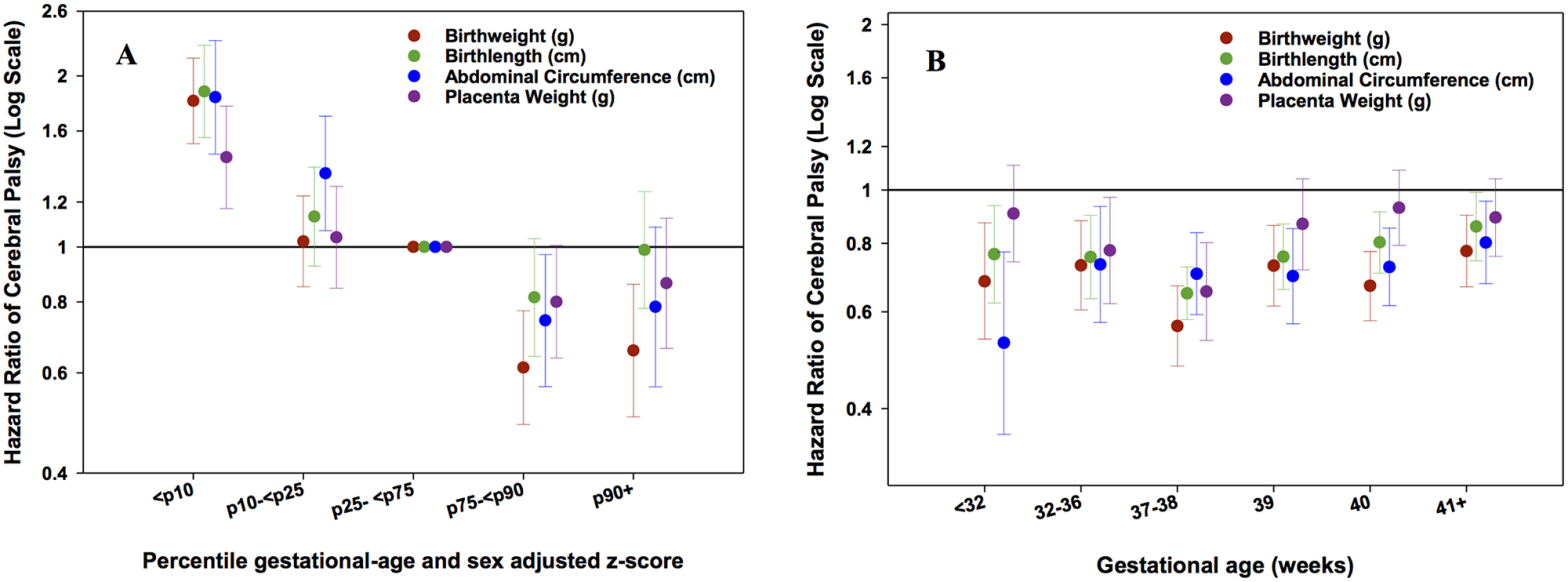
A: Hazard Ratios (HR) for CP according to 5 percentile groups (≤10, >10–25, >25–75 (reference), >75–90, >90 percentile) of sex and gestational age adjusted z-scores for birth weight, birth length, abdominal circumference, and placenta weight in all subjects, and Fig 1B. across strata of gestational age (<32, 32–36, 37–38, 39, 40, ≥41 weeks). Footnote: Models were adjusted for maternal age, paternal age, smoking, first liveborn, parents’ education, year of child’s birth, vaginal bleeding, diabetes in pregnancy, hypertensive disorder during pregnancy and placenta disorders.

The association with CP for different percentile levels of z-score for birth weight, birth length, abdominal circumference and placental weight showed similar results (Fig 1A, S4 Table). Low z-scores were positively associated with CP for all measurements while a high z-score for birth weight was significantly negatively associated with CP. Fig 1B (S5 Table) shows that the negative association between these factors and CP was independent of gestational age strata.

### Head Circumference

**Fig 2:**
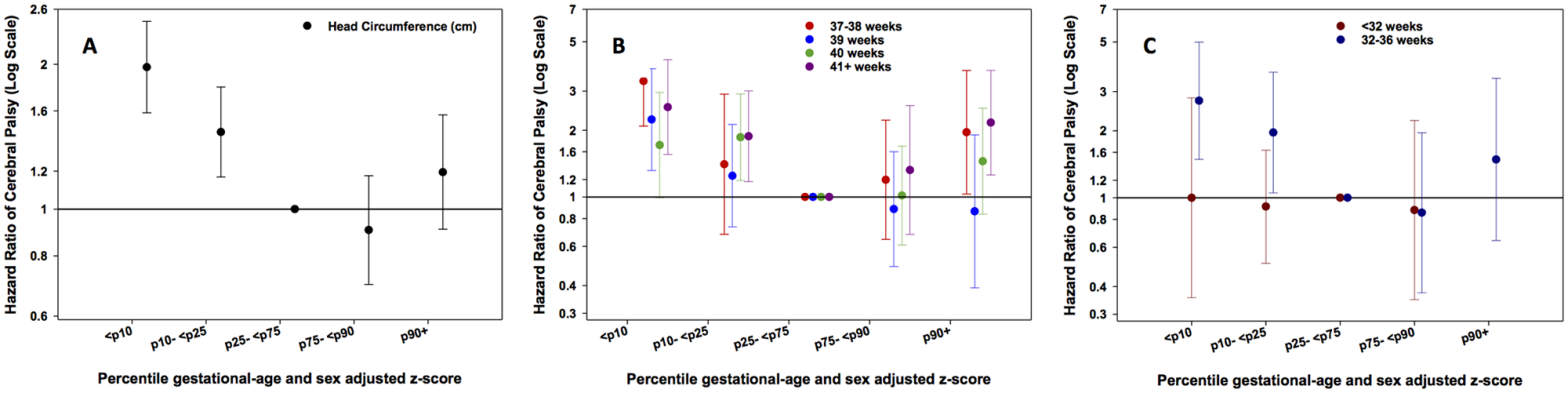
A: Hazard Ratios (HR) for CP according to 5 percentile groups (≤10, >10–25, >25–75 (reference), >75–90, >90 percentile) of sex and gestational age adjusted z-scores for head circumference in all subjects, Fig 2B. in subgroups of term and post-term gestational age (37–38, 39, 40, ≥41 weeks), and Fig 2C. in subgroups of preterm gestational age (<32, 32–36 weeks). Footnote: Models were adjusted for maternal age, paternal age, smoking, first liveborn, parents’ education, year of child’s birth, vaginal bleeding, diabetes in pregnancy, hypertensive disorder during pregnancy and placenta disorders.

The associations of head circumference z-scores with CP according to percentile level (Fig 2A and S4 Table) and according to gestational age (Fig 2B and 2C, S6 Table) strata are shown. The data show that, with the exception of very preterm infants (< 32 weeks), low z scores of head circumference are associated with CP. Children with higher z scores (>90^th^ percentile) of head circumference also showed a trend toward an association with higher risk of CP, but this “U-shaped” association was only significant for children born at 37–38 or 41+ weeks.

### Ponderal Index

**Fig 3:**
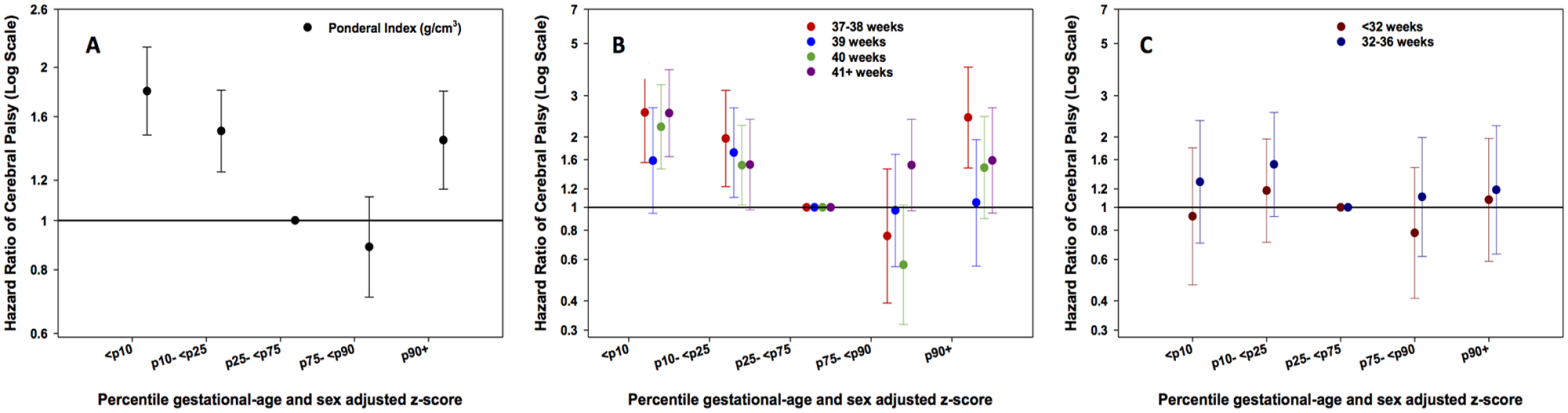
A: Hazard Ratios (HR) for CP according to 5 percentile groups (≤10, >10–25, >25–75 (reference), >75–90, >90 percentile) of sex and gestational age adjusted z-scores for ponderal index in all subjects, Fig 3B. in subgroups of term and post-term gestational age (37–38, 39, 40, ≥41 weeks), and Fig 3C. in subgroups of preterm gestational age (<32, 32–36 weeks). Footnote: Models were adjusted for maternal age, paternal age, smoking, first liveborn, parents’ education, year of child’s birth, vaginal bleeding, diabetes in pregnancy, hypertensive disorder during pregnancy and placenta disorders.

Table 2 shows that the ponderal index, measured as a continuous z-score, was not significantly associated with CP in adjusted models. However, Fig 3A (S4 Table) shows that the association of the ponderal index with CP is “U shaped” when examined in percentile groups of z-scores, where both low (below the 25^th^ percentile) and high (above the 90^th^ percentile) z-scores for this index are positively associated with CP. This U-shaped trend was shown in term and post-term gestational age strata (Fig 3B, S7 Table), however children born preterm and very preterm (Fig 3C, S7 Table) showed no association between z-score percentiles for ponderal index and CP.

### Head/Abdominal Circumference and Cephalization Index

**Fig 4:**
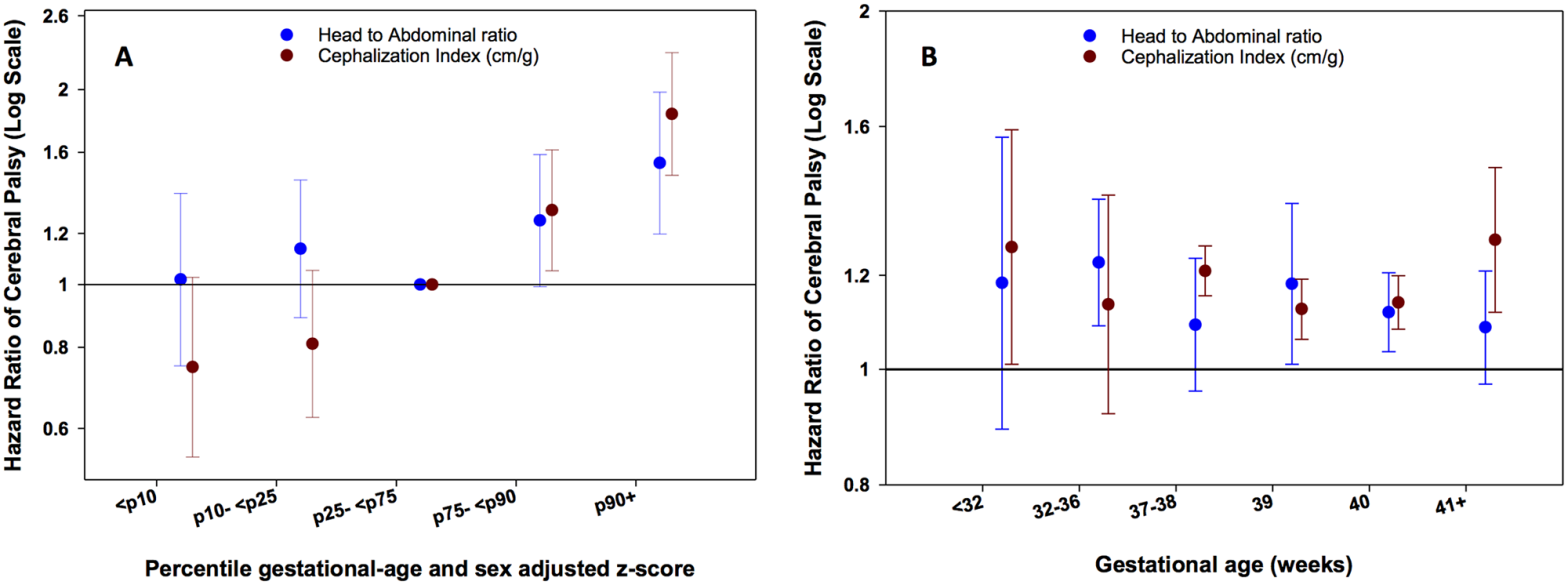
A: Hazard Ratios (HR) for CP according to 5 percentile groups (≤10, >10–25, >25–75 (reference), >75–90, >90 percentile) of sex and gestational age adjusted z-scores for head/abdominal circumference ratio and cephalization index in all subjects, and Fig 4B. across strata of gestational age (<32, 32–36, 37–38, 39, 40, ≥41 weeks). Footnote: Models were adjusted for maternal age, paternal age, smoking, first liveborn, parents’ education, year of child’s birth, vaginal bleeding, diabetes in pregnancy, hypertensive disorder during pregnancy and placenta disorders.

The continuous z-scores for head/abdominal circumference and cephalization index were positively and significantly associated with CP (Table 2). Higher percentiles of z-scores (>75^th^ percentile) for both indices were also associated with higher risk of CP (Fig 4A and S4 Table). Furthermore, associations with CP of continuous z-scores for these indices were similar across strata of gestational age (Fig 4B and S5 Table).

### Birth Weight/Placenta Weight Ratio

**Fig 5:**
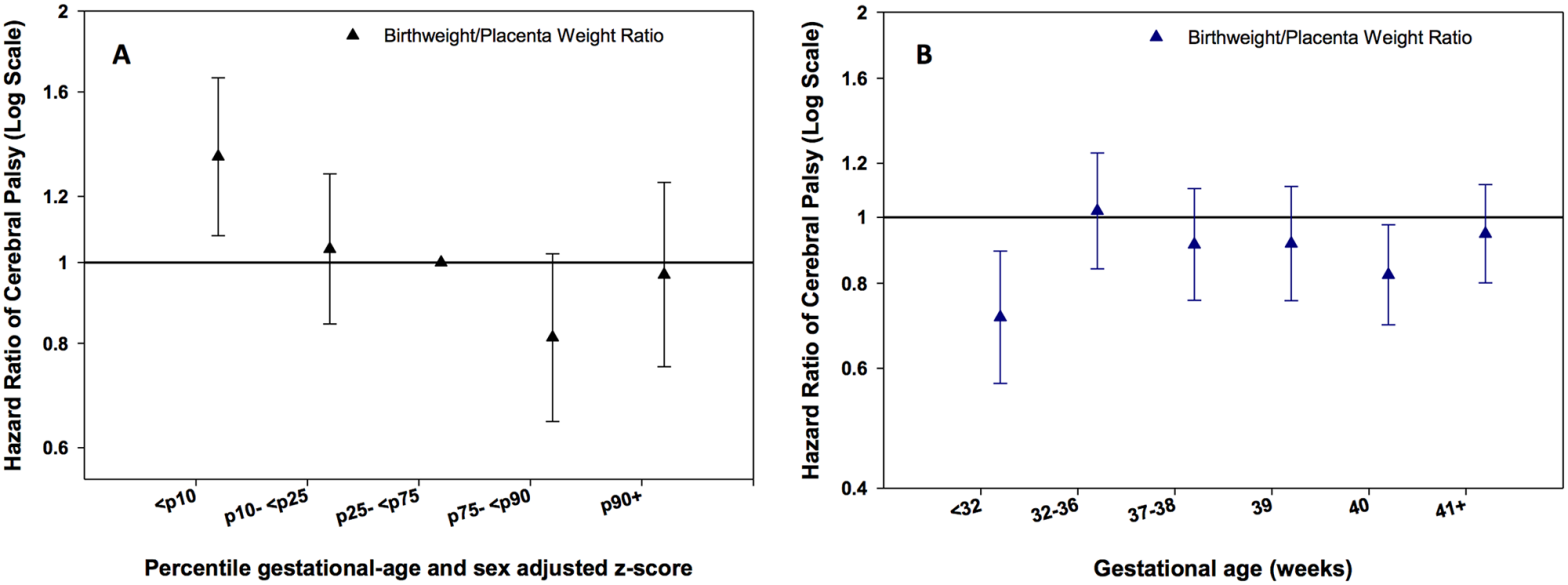
A: Hazard Ratios (HR) for CP according to 5 percentile groups (≤10, >10–25, >25–75 (reference), >75–90, >90 percentile) of sex and gestational age adjusted z-scores for birth weight/placenta weight ratio in all subjects, in all subjects, and Fig 5B. across strata of gestational age (<32, 32–36, 37–38, 39, 40, ≥41 weeks). Footnote: Models were adjusted for maternal age, paternal age, smoking, first liveborn, parents’ education, year of child’s birth, vaginal bleeding, diabetes in pregnancy, hypertensive disorder during pregnancy and placenta disorders.

A higher birth weight-placenta weight ratio z-score was negatively and significantly associated with CP (Table 2). This association is mostly explained by the increased risk of CP for children with a ratio ≤ 10^th^ percentile (Fig 5A). Negative associations of continuous z-scores for birth weight/placenta weight ratio with CP were similar across strata of gestational age, but only significant for children born at < 32 weeks and 40 weeks (Fig 5B).

## Discussion

This study shows that reduced fetal growth, as seen in different indices of fetal size and disproportionate growth, is associated with CP. In some instances, large fetal size was also associated with CP. Notably, each measurement of fetal growth examined in this study was associated with CP in a specific manner, suggesting that different underlying mechanisms and potential prenatal etiologic pathways leading to CP may be reflected in the different measures.

Ponderal index is an index of leanness of the newborn. Asymmetric fetal growth, characterized by a low ponderal index, presumably reflects fetal malnutrition[39] and has therefore received wide medical attention. Infants born with “asymmetry” defined this way have higher rates of perinatal morbidity[40] and mortality.[41] In our study low weight, low length and low ponderal index were associated with CP, which corroborates previous findings. However, high ponderal index in our study was also associated with higher risk of CP. Infants born with ponderal index above the 90^th^ percentile (large weight for length) had a 44% increased risk of CP (HR: 1.44, 95%CI: 1.15–1.80).

The scientific literature concerning the association of ponderal index with CP is conflicting. In 1992, Blair and Stanley reported that 23% of the children with CP have a low ponderal index.[42] Subsequently, Williams et al reported an association between CP and low ponderal index in infants born with low Apgar score[43]. In the same year, however, a larger Danish study[44] found no significant correlation between CP and ponderal index. In a recent study, including only term deliveries, Dahlseng et al found a strong linear negative association between ponderal index and the risk of CP.[45] They additionally found that children with the greatest body length and largest head circumference but with a low ponderal index had an excess risk of spastic quadriplegic and dyskinetic CP types. Since these types of CP were reported to be associated with perinatal trauma[46] the authors concluded that CP in children who were large at birth is more likely to be due to intrapartum factors.

Head Circumference was observed to be positively associated with CP when low, as well as when high confirming findings previously reported by Blair and Stanley.[42]. In addition, children with CP had heads that were disproportionately larger than expected for their given birth weight or abdominal circumference, irrespective of gestational age. The “u-shaped” association with CP seen with head circumference alone was not evident in head/abdominal circumference and cephalization indices. In these indices only higher ratio measures were associated with increased risk of CP. Asymmetric growth, defined as disproportionately large head to abdominal circumference ratio[47], is attributed to the capacity of the fetus to adapt and redistribute its cardiac output in favor of vital organs[48] Children with this type of asymmetric growth in a previous study had a higher risk of suboptimal neuromotor development at 9–15 weeks[49] and short-term memory difficulties at nine years of age[50]. Asymmetric growth defined by the cephalization index was reported to be a useful predictor of neurodevelopmental outcome in children with intrauterine growth restriction[51, 52], and also shown to be possibly associated with CP[53]. Asymmetry in ratios of growth measurements involving head circumferences, however, could also be attributed to other causes such as hydrocephaly. Fetal growth impairment is most likely a part of the prenatal disease process leading to CP. Disproportionate growth is perhaps a stronger indicator because it is more closely linked to pathology. A part of those with proportional low growth indicators may be small by nature.

Birth weight/ placenta weight ratio has been used as an indicator of placental function[6, 37]. Our data showed an overall negative association between CP and birth weight-placental weight ratio, with the lowest negative point estimate in very preterm infants (< 32 weeks). Children with CP, in particular those born very preterm, had a lower birth weight compared to what was expected given the size of their placenta. Birth weight and placental weight are strongly correlated[54] and small for gestational age infants have smaller placentae than normal weight controls[55]. Low birth weight adjusted for gestational age is a known strong risk indicator for CP[56].

The strength of the study is the fact that CP was ascertained by the strict inclusion criteria of the Danish National Cerebral Palsy Registry and the large size of the cohort including a large number CP cases. An additional strength is that data on measurements at birth were collected from the Danish National Birth Registry, which records measurements on all births in Denmark. Therefore, underreporting or selection bias is unlikely to occur since medical care is free of charge for residents in Denmark.

Our study is limited by the amount of missing data for maternal smoking and parental education. We used multiple imputation methods to fill in missing values, but because of the large number of missing values, imputation may have led to some loss of precision in the estimates and bias to the null. However, sensitivity analysis using patients with complete data showed similar results. Additionally, although we used education as a marker of socio-economic status, there may still be residual confounding by socio-economic status that is not represented with this variable. Other unknown confounders may also be present and contribute to residual confounding. By conditioning on gestational age we may induce collider stratification bias[57] if we do not adjust for all factors leading to low gestational age and CP. That is, we may induce an erroneous statistical relationship between otherwise independent factors, such as fetal growth measurements and CP, by adjusting for gestational age, if gestational age and CP shared a common cause.

Our study was restricted to children who were born and then survived and did not emigrate from Denmark prior to the age of 1 year; Danish emigration rates are low and unlikely to be a source of selection bias. Another limitation of our study is the potential lack of generalizability to Non-Caucasians due to the lack of racial-ethnic diversity of the Danish population. Based on our study data we cannot pinpoint the timing or underlying cause of insult or the timing of initiation of disproportionate growth associated with CP, since the events (insult and resulting growth changes) could arise at various points in pregnancy. In addition, the study is limited by the absence of imaging or other information that would permit further characterization of the nature of the problems underlying aberrant anthropometric measurements. The biology of growth, body and brain development is highly complex and incompletely understood. Further studies based on longitudinal recording of fetal growth are needed to determine if measurements of asymmetric growth could have predictive value in determining CP outcomes. Given this lack of knowledge, a proper analytical strategy cannot be well identified. Conditioning on intermediate variables may not only block the causal path we take an interest in, but may also produce,[57]-stratification bias; a problem not only of course in this study, but in most other studies of factors related to reproduction and CP.[58]

In conclusion we observed that risk of CP was associated with reduced fetal growth measured in different dimensions; smaller anthropometric measures overall as well as lower weight for length, lower weight for placental weight, lower weight relative to head size.. In some instances, greater fetal growth (large heads, large weight for length) was associated with CP also. These results support the concept that CP is a condition arising from pre and perinatal conditions that, in some cases, contribute to disproportionate fetal growth, perhaps even early in fetal life.

## Supporting Information

S1 Fig Cohort Construction. (TIF)

S1 Table ICD-10 Codes Used to Define Maternal disorders of Pregnancy. (DOC)

S2 Table Median and Interquartile range (25^th^ and 75^th^ percentile) for sex and gestational age adjusted z-scores of exposure variables according to presence of CP in the infant. (DOC)

S3 Table Complete Case Analyses Hazard Ratios (HR) for CP according to continuous measurements of sex and gestational age adjusted z-scores for newborn anthropometric measures and indices (all subjects). (DOC)

S4 Table Hazard Ratios (HR) for CP according to 5 percentile groups of sex and gestational age adjusted z-scores for newborn anthropometric measures and indices (all subjects). (DOC)

S5 Table Hazard Ratios (HR) for CP according to 5 percentile groups of sex and gestational age adjusted z-scores for newborn anthropometric measures and indices (all subjects) in subgroups of weeks of gestational age. (DOC)

S6 Table Hazard Ratios (HR) for CP according to 5 percentile groups of sex and gestational age adjusted z-scores for head circumference in subgroups of weeks of gestational age. (DOC)

S7 Table Hazard Ratios (HR) for CP according to 5 percentile groups of sex and gestational age adjusted z-scores for ponderal index in subgroups of weeks of gestational age. (DOC)
