# Supplementary material for: Disproportionate Fetal Growth and the Risk for Congenital Cerebral Palsy in Singleton Births
Source: PLoS One. 2015 May 14;10(5):e0126743. doi: 10.1371/journal.pone.0126743 (PMC4431832; doi:10.1371/journal.pone.0126743)
Supplement: S1 Table — (DOC) [file pone.0126743.s002.doc]

**S2 Table**: ICD-10 Codes Used to Define Maternal disorders of Pregnancy

| **Condition** | **ICD-10 Codes** |
| --- | --- |
| Hypertensive disorder of pregnancy | O11, O13- O15 |
| Diabetes during pregnancy | O24 |
| Vaginal bleeding during pregnancy | O47, O67 |
| Placenta disorders | O365, O411D, O43-45, O72-O73 |
