# Supplementary material for: Disproportionate Fetal Growth and the Risk for Congenital Cerebral Palsy in Singleton Births
Source: PLoS One. 2015 May 14;10(5):e0126743. doi: 10.1371/journal.pone.0126743 (PMC4431832; doi:10.1371/journal.pone.0126743)
Supplement: S2 Table — (DOC) [file pone.0126743.s003.doc]

**S3 Table: Median and Interquartile range (25^th^ and 75^th^** percentile) for sex and gestational age adjusted z-scores of exposure variables according to presence of CP in the infant

|  |  |  | |  | |
| --- | --- | --- | --- | --- | --- |
|  |  | **Non-CP n=502,801** | | **CP n=983** | |
|  |  | Median | (interquartile range) | Median | (interquartile range) |
| **Birth weight** | | -0.03 | (-0.66,0.62) | -0.30 | (-0.97,0.28) |
| **Birth length** | | -0.01 | (-0.69,0.69) | -0.23 | (-0.91,0.48) |
| **Head Circumference** | | -0.02 | (-0.65,0.63) | -0.31 | (-1.04,0.43) |
| **Abdominal Circumference** | | 0 | (-0.62,0.57) | -0.36 | (-0.92,0.31) |
| **Placental Weight** | | -0.09 | (-0.68,0.59) | -0.29 | (-0.87,0.41) |
| **Ponderal Index** | | -0.05 | (-0.54,0.48) | -0.21 | (-0.77,0.41) |
| **Cephalization Index** | | -0.09 | (-0.66,0.55) | 0.13 | (-0.46,0.98) |
| **Head-Abd. Circ. Ratio** | | -0.03 | (-0.58,0.54) | 0.07 | (-0.53,0.77) |
| **Birthweight/placenta ratio** | | -0.08 | (-0.66,0.57) | -0.19 | (-0.8,0.49) |

CP: congenital cerebral palsy, Head-Abd. Circ. Ratio: Head-Abdominal Circumference Ratio
