# Supplementary material for: Disproportionate Fetal Growth and the Risk for Congenital Cerebral Palsy in Singleton Births
Source: PLoS One. 2015 May 14;10(5):e0126743. doi: 10.1371/journal.pone.0126743 (PMC4431832; doi:10.1371/journal.pone.0126743)
Supplement: S3 Table — (DOC) [file pone.0126743.s004.doc]

| **S4 Table: Complete Case Analyses Hazard Ratios (HR) for CP according to continuous measurements of sex and gestational age adjusted z-scores for newborn anthropometric measures and indices (all subjects)** | | | | | | |
| --- | --- | --- | --- | --- | --- | --- |
|  | **N without CP** | **N with CP** | **HR** | **aHR*** | **95%CI** | |
| **Birth weight** | 286,232 | 538 | 0.69 | 0.72 | (0.66 | 0.79) |
| **Birth length** | 284,078 | 488 | 0.76 | 0.78 | (0.72 | 0.84) |
| **Head Circumference** | 248,776 | 385 | 0.78 | 0.79 | (0.72 | 0.87) |
| **Abdominal Circumference** | 241,688 | 347 | 0.71 | 0.76 | (0.68 | 0.84) |
| **Placental Weight** | 245,480 | 456 | 0.84 | 0.80 | (0.72 | 0.88) |
| **Ponderal Index** | 284,047 | 487 | 0.92 | 0.93 | (0.84 | 1.04) |
| **Cephalization Index** | 248,246 | 383 | 1.15 | 1.15 | (1.11 | 1.19) |
| **Head-Abd. Circ. Ratio** | 241,402 | 342 | 1.12 | 1.10 | (1.03 | 1.17) |
| **Birth weight/placenta ratio** | 244,796 | 447 | 0.88 | 0.97 | (0.89 | 1.07) |

CP: congenital cerebral palsy, HR: Hazard Ratio, aHR: adjusted hazard ratio, CI: confidence interval

Head-Abd. Circ. Ratio: Head-Abdominal Circumference Ratio

All exposures were analyzed as sex and gestational adjusted z-scores.
Models were adjusted for maternal age, paternal age, smoking, first liveborn, parents’ education, year of child’s birth,
vaginal bleeding, diabetes in pregnancy, hypertensive disorder during pregnancy and placenta disorders.
