# Supplementary material for: Disproportionate Fetal Growth and the Risk for Congenital Cerebral Palsy in Singleton Births
Source: PLoS One. 2015 May 14;10(5):e0126743. doi: 10.1371/journal.pone.0126743 (PMC4431832; doi:10.1371/journal.pone.0126743)
Supplement: S4 Table — (DOC) [file pone.0126743.s005.doc]

**S5 Table: Hazard Ratios (HR) for CP according to 5 percentile groups of sex and gestational age adjusted z-scores for newborn anthropometric measures and indices (all subjects)**

|  | Percentile of sex and gestational age adjusted z-score | | | | |
| --- | --- | --- | --- | --- | --- |
|  | <p10 | p10-<p25 | p25-<p75 | p75-<p90 | p90+ |
|  | aHR (95%CI) | aHR (95%CI) | aHR (95%CI) | aHR (95%CI) | aHR (95%CI) |
| **Birth weight** | 1.81 (1.52, 2.15) | 1.02 (0.85, 1.23) | 1 (reference) | 0.61 (0.49, 0.77) | 0.66 (0.50, 0.86) |
| **Birth length** | 1.88 (1.56, 2.26) | 1.13 (0.93, 1.38) | 1 (reference) | 0.82 (0.64, 1.04) | 0.99 (0.78, 1.25) |
| **Head Circumference** | 1.97 (1.59, 2.45) | 1.45 (1.17, 1.79) | 1 (reference) | 0.91 (0.70, 1.17) | 1.19 (0.91, 1.57) |
| **Abdominal Circumference** | 1.84 (1.46, 2.31) | 1.35 (1.07, 1.70) | 1 (reference) | 0.74 (0.57, 0.97) | 0.78 (0.57, 1.08) |
| **Placental Weight** | 1.44 (1.17, 1.77) | 1.04 (0.85, 1.28) | 1 (reference) | 0.80 (0.64, 1.01) | 0.86 (0.66, 1.12) |
| **Ponderal Index** | 1.80 (1.47, 2.19) | 1.50 (1.25, 1.80) | 1 (reference) | 0.89 (0.71, 1.11) | 1.44 (1.15, 1.80) |
| **Cephalization Index** | 0.75 (0.54, 1.03) | 0.81 (0.62, 1.05) | 1 (reference) | 1.30 (1.05, 1.62) | 1.83 (1.48, 2.28) |
| **Head-Abd. Circ. Ratio** | 1.02 (0.75, 1.38) | 1.14 (0.89, 1.45) | 1 (reference) | 1.26 (0.99, 1.59) | 1.54 (1.20, 1.98) |
| **Birth weight/placenta ratio** | 1.34 (1.08, 1.66) | 1.04 (0.84, 1.28) | 1 (reference) | 0.81 (0.65, 1.02) | 0.97 (0.75, 1.25) |

CP: congenital cerebral palsy, aHR: adjusted hazard ratio, CI: confidence interval
Head-Abd. Circ. Ratio: Head-Abdominal Circumference Ratio

All exposures were analyzed as sex and gestational adjusted z-scores.
Models were adjusted for maternal age, paternal age, smoking, first liveborn, parents’ education, year of child’s birth,
vaginal bleeding, diabetes in pregnancy, hypertensive disorder during pregnancy and placenta disorders
