# Supplementary material for: Disproportionate Fetal Growth and the Risk for Congenital Cerebral Palsy in Singleton Births
Source: PLoS One. 2015 May 14;10(5):e0126743. doi: 10.1371/journal.pone.0126743 (PMC4431832; doi:10.1371/journal.pone.0126743)
Supplement: S5 Table — (DOC) [file pone.0126743.s006.doc]

**S6 Table: Hazard Ratios (HR) for CP according to 5 percentile groups of sex and gestational age adjusted z-scores for newborn anthropometric measures and indices (all subjects) in subgroups of weeks of gestational age.**

|  | Gestational Age (Weeks) | | | | | |
| --- | --- | --- | --- | --- | --- | --- |
|  | <32 | 32-36 | 37-38 | 39 | 40 | 41+ |
|  | aHR (95%CI) | aHR (95%CI) | aHR (95%CI) | aHR (95%CI) | aHR (95%CI) | aHR (95%CI) |
| **Birth weight** | 0.68 (0.54, 0.87) | 0.73 (0.61, 0.88) | 0.56 (0.48, 0.67) | 0.73 (0.62, 0.86) | 0.67 (0.58, 0.77) | 0.77 (0.67, 0.90) |
| **Birth length** | 0.76 (0.62, 0.94) | 0.76 (0.63, 0.90) | 0.65 (0.58, 0.72) | 0.76 (0.66, 0.87) | 0.80 (0.71, 0.91) | 0.86 (0.74, 0.99) |
| **Abdominal Circumference** | 0.53 (0.36, 0.77) | 0.73 (0.57, 0.93) | 0.70 (0.59, 0.84) | 0.70 (0.57, 0.85) | 0.72 (0.62, 0.85) | 0.80 (0.68, 0.95) |
| **Placental Weight** | 0.91 (0.74, 1.11) | 0.78 (0.62, 0.97) | 0.65 (0.53, 0.80) | 0.87 (0.72, 1.05) | 0.93 (0.79, 1.09) | 0.89 (0.76, 1.05) |
| **Cephalization Index** | 1.27 (1.01, 1.59) | 1.13 (0.92, 1.40) | 1.21 (1.15, 1.27) | 1.12 (1.06, 1.19) | 1.14 (1.08, 1.20) | 1.29 (1.12, 1.48) |
| **Head-Abd. Circ. Ratio** | 1.18 (0.89, 1.57) | 1.23 (1.09, 1.39) | 1.09 (0.96, 1.24) | 1.18 (1.01, 1.38) | 1.12 (1.04, 1.21) | 1.09 (0.97, 1.21) |
| **Birth weight/placenta ratio** | 0.71 (0.57, 0.89) | 1.02 (0.84, 1.24) | 0.91 (0.76, 1.10) | 0.92 (0.75, 1.11) | 0.82 (0.69, 0.98) | 0.95 (0.80, 1.12) |

CP: congenital cerebral palsy, aHR: adjusted hazard ratio, CI: confidence interval
Head-Abd. Circ. Ratio: Head-Abdominal Circumference Ratio

All exposures were analyzed as sex and gestational adjusted z-scores.
Models were adjusted for maternal age, paternal age, smoking, first liveborn, parents’ education, year of child’s birth,
vaginal bleeding, diabetes in pregnancy, hypertensive disorder during pregnancy and placenta disorders
