# Supplementary material for: Disproportionate Fetal Growth and the Risk for Congenital Cerebral Palsy in Singleton Births
Source: PLoS One. 2015 May 14;10(5):e0126743. doi: 10.1371/journal.pone.0126743 (PMC4431832; doi:10.1371/journal.pone.0126743)
Supplement: S6 Table — (DOC) [file pone.0126743.s007.doc]

**S7 Table: Hazard Ratios (HR) for CP according to 5 percentile groups of sex and gestational age adjusted z-scores for** head circumference in subgroups of weeks of gestational age.

|  | Head Circumference | | | | |
| --- | --- | --- | --- | --- | --- |
|  | Percentile of sex and gestational age adjusted z-score | | | | |
|  | <p10 | p10-<p25 | p25-<p75 | p75-<p90 | p90+ |
| Gestational Age (Weeks) | aHR (95%CI) | aHR (95%CI) | aHR (95%CI) | aHR (95%CI) | aHR (95%CI) |
| <32 | 1.00 (0.36, 2.80) | 0.91 (0.51, 1.64) | 1 (reference) | 0.88 (0.35, 2.22) | 0.09 (0.12, 0.63) |
| 32-36 | 2.73 (1.49, 4.99) | 1.96 (1.05, 3.66) | 1 (reference) | 0.86 (0.38, 1.96) | 1.49 (0.64, 3.44) |
| 37-38 | 3.33 (2.09, 5.31) | 1.41 (0.68, 2.91) | 1 (reference) | 1.20 (0.65, 2.22) | 1.96 (1.03, 3.72) |
| 39 | 2.23 (1.32, 3.78) | 1.25 (0.73, 2.12) | 1 (reference) | 0.88 (0.49, 1.60) | 0.86 (0.39, 1.91) |
| 40 | 1.72 (0.99, 2.96) | 1.86 (1.19, 2.91) | 1 (reference) | 1.02 (0.61, 1.70) | 1.45 (0.84, 2.51) |
| 41+ | 2.54 (1.56, 4.15) | 1.88 (1.17, 3.01) | 1 (reference) | 1.32 (0.68, 2.58) | 2.16 (1.26, 3.72) |

CP: congenital cerebral palsy, aHR: adjusted hazard ratio, CI: confidence interval

Models were adjusted for maternal age, paternal age, smoking, first liveborn, parents’ education, year of child’s birth,
vaginal bleeding, diabetes in pregnancy, hypertensive disorder during pregnancy and placenta disorders.
